# Heart Disease, Blood Pressure and the Nauheim-Schott Treatment

**Published:** 1910-06

**Authors:** 


**Heart Disease, Blood Pressure and the Nauheim-Schott Treatment.** By **Louis Faugeres Bishop,** A.M., M.D., Clinical Professor of Heart and Circulatory Diseases, Fordham University, School of Medicine, New York. Third edition. 12 mo, pp. 284. Illustrated. New York: E. B. Treat & Co. 1909. (Cloth, $3.00.)

The exhaustion of the second edition and a call for a third, has afforded the author an opportunity to review and bring up to date his observation and conclusions relative to the Schott-Nauheim treatment of diseases of the circulatory system. The subject is ably presented and will open up a new field of thought to the reader, enabling him to more successfully cope with the diseases considered. J. A. R.

